# Effects of Epithelial to Mesenchymal Transition on T Cell Targeting of Melanoma Cells

**DOI:** 10.3389/fonc.2014.00367

**Published:** 2014-12-17

**Authors:** Katherine Woods, Anupama Pasam, Aparna Jayachandran, Miles C. Andrews, Jonathan Cebon

**Affiliations:** ^1^Cancer Immunobiology Laboratory, Ludwig Institute for Cancer Research, Melbourne-Austin Branch, Olivia Newton John Cancer and Wellness Centre, Melbourne, VIC, Australia; ^2^School of Cancer Medicine, La Trobe University, Melbourne, VIC, Australia

**Keywords:** T-lymphocytes, melanoma, epithelial–mesenchymal transition, tumor antigens, T cell killing, cancer testis antigens

## Abstract

Melanoma cells can switch phenotype in a manner similar to epithelial to mesenchymal transition (EMT). In this perspective article, we address the effects of such phenotype switching on T cell targeting of tumor cells. During the EMT-like switch in phenotype, a concomitant change in expression of multiple tumor antigens occurs. Melanoma cells undergoing EMT escape from killing by T cells specific for antigens whose expression is downregulated by this process. We discuss melanoma antigens whose expression is influenced by EMT. We assess the effect of changes in the expressed tumor antigen repertoire on T-cell mediated tumor recognition and killing. In addition to escape from T cell immunity via changes in antigen expression, mesenchymal-like melanoma cells are generally more resistant to classical chemotherapy and radiotherapy. However, we demonstrate that when targeting antigens whose expression is unaltered during EMT, the capacity of T cells to kill melanoma cell lines *in vitro* is not influenced by their phenotype. When considering immune therapies such as cancer vaccination, these data suggest escape from T cell killing due to phenotype switching in melanoma could potentially be avoided by careful selection of target antigen.

## Introduction

The ability of melanoma cells to switch phenotype from proliferative to more invasive states, in a process similar to classical epithelial-mesenchymal transition (EMT), has been well described (1). Non-motile, polarized, and proliferative epithelial-like (E-like) cells, acquire motile, fibroblast-like mesenchymal characteristics (2). The process contributes to the heterogeneity of the tumor, and may be a factor in disease progression (3, 4). In melanoma, the EMT-like switch in phenotype can be driven by environmental pressures such as changes in the cytokine milieu at the tumor site, e.g., due to inflammation (5), and drug treatments such as inhibition of oncogenic BRAF (6).

We have previously generated a panel of 57 melanoma cell lines derived from patient samples (7). We have characterized these cell lines as E-like or mesenchymal-like (M-like) on the basis of expression levels of E-cadherin or N-cadherin, respectively (8). Between these two cohorts, we documented significant disparity in gene expression levels of many mRNA, reflective of the functional and phenotypic differences between the groups. Included in these differentially expressed genes, were mRNAs encoding melanocytic differentiation proteins, such as Melan-A and tyrosinase. Both of these have demonstrated antigenic epitopes, which have been previously used as targets of cancer vaccines in melanoma clinical trials (9–11), and are currently the target antigens in ongoing trials (NCT01331915, NCT01748747). Expression of these genes, as well as the master regulator of differentiation, MITF (Microphthalmia-associated transcription factor), was significantly downregulated in M-like cell lines compared to E-like (8). A decrease in expression of the melanoma differentiation antigens (MDA) under the control of MITF during EMT has also been reported by others, e.g., gp100.

The cancer testis group of antigens (CTAg) have been identified as important immune targets in melanoma, as well as other cancer types, and have been studied extensively (12, 13). Expression of CT genes is generally restricted to immune-privileged body sites, yet is frequently and selectively re-activated in a broad variety of human cancers (12). Almost 150 families have been defined (http://www.cta.lncc.br) (14). Their immunogenicity and selective expression in cancer makes them attractive targets for therapeutic cancer vaccines, and many have been used for this purpose over the past ~2 decades, with limited success (15). In parallel with EMT-associated downregulation of certain melanoma antigens, particularly the differentiation antigens, which are under the control of MITF, we have also observed upregulation in expression of a number of cancer testis antigens (CTAg). In particular, we showed that expression of the SPANX family of CTAg was enhanced in M-like compared to E-like cell lines (8). This group of CTAg has documented immunogenicity (16) and their expression is found at higher levels in more aggressive tumors (17). A range of CTAg (including SPANX and a number of MAGE family members) were also recently shown to be upregulated following EMT in a colorectal cancer cell line (18).

Independent of the well-documented functional differences between E- and M-like cells, the changes in expression of immunogenic antigens between melanoma phenotypic subsets has profound implications for immune-based recognition and clearance of tumor. For example, based on our studies, immune responses induced against antigens such as Melan-A and tyrosinase would potentially only be effective in clearing differentiated E-like tumor cells. Indeed, a previous study highlighted the EMT-like phenotypic switch in melanoma as a method of escape from adoptive T cell therapy with T-lymphocytes targeting Melan-A (19). This study also showed that M-like cells were poorly recognized by T-lymphocytes that were specific for melanocytic antigens, whereas recognition by T-lymphocytes specific for non-melanocytic antigens was unaffected or even increased *in vivo*.

Taken together, the studies discussed here highlight the disparity of the immunogenic profile of E-like versus M-like tumor cells, and emphasize the importance of appropriate target antigen selection for cancer vaccine/T cell targeting approaches.

The M-like phenotype has been associated with tumor progression, invasion, and metastasis in melanoma (20, 21). This generally more aggressive phenotype is accompanied by acquisition of resistance to drug treatment and radiotherapy (22). Furthermore, this phenotype also emerges after the development of resistance against BRAF inhibitors (6). Thus, M-like cells are generally considered more difficult to eradicate, by multiple mechanisms, compared with their E-like counterparts.

In this article, we ask whether M-like melanoma cells are inherently resistant to killing by T-lymphocytes at a functional level, reflective of the mesenchymal state, or whether an escape from T cell-mediated killing via phenotype switching is simply reflective of the decrease in expression of many target tumor antigens. We show that expression of the prototypic cancer testis antigen NY-ESO-1 is unaltered between melanoma phenotypic states. We compare the ability of NY-ESO-1 or Melan-A specific T cell clones to recognize and kill melanoma cell lines, which were phenotypically either E-like or switched to M-like following incubation with TGFβ (23).

## Materials and Methods

### Melanoma cell lines and culture

Establishment and characterization of the melanoma cell lines used has been previously described (7). Cells were cultured in RPMI 1640, 2 mM Glutamax, 100 IU/ml Penicillin, 100 μg/ml Streptomycin, and 10% fetal calf serum (RF10) (all Invitrogen). To induce EMT, subconfluent cell lines were incubated with 5 ng/ml TGF-beta 1 (Pepro Tech) for 72 h.

### Gene expression array

The gene expression array method and analysis have been previously described (24). Briefly, genomic DNA was purified from melanoma cell lines (Qiagen AllPrep kits) and assayed using Illumina standard protocols.

### Quantitative real-time polymerase cell reaction (qRT-PCR)

cDNA was generated using the High Capacity cDNA Reverse Transcription Kit (Applied Biosystems). Quantitative polymerase chain reaction (qPCR) was performed using the QuantiFast SYBR Green PCR Kit (Qiagen). Primer sequences were as follows: NY-ESO-1 (forward) 5′-gagccgcctgcttgagtt-3′ and (reverse) 5′-agcactgcgtgatccacatc-3′; Melan-A (forward) 5′-gagaaaaactgtgaacctgtggt-3′ and (reverse) 5′-gactgttctgcagagagtttctcat-3′; N-cadherin (forward) 5′-ctccatgtgccggatagc-3′ and (reverse) 5′-cgatttcaccagaagcctctac-3′ β-actin (forward) 5′-ctggaacggtgaaggtgaca-3′ and (reverse) 5′-cggccacattgtgaactttg-3′.

### Immunofluorescence

Melanoma cell lines were treated with 5 ng/ml TGF-β1 for 72 h and fixed with 4% paraformaldehyde. Mouse anti-E-cadherin antibody (HECD1, Invitrogen) and rabbit anti-N-cadherin antibody (AB18203, Abcam) were applied at 2 and 1 μg/ml concentration, respectively, overnight at 4°C. Cells were subsequently treated with Alexa flour 488 (anti-mouse) and 555 (anti-rabbit) conjugated secondary antibodies for 45 min at room temperature (Molecular probes, USA). Cells were counter stained with DAPI for 10 min.

### Generation of antigen specific CD8^+^ T cell clones

CD8^+^ T cell clones specific for the NY-ESO-1 HLA-A*0201 restricted peptide 157–165, or the Melan-A HLA-A*0201 restricted peptide 26–35, were generated from patients who consented to participate in our cancer research protocol (approved by Austin Health Human Research Ethics Committee). PBMC (peripheral blood mononuclear cells) were stimulated with 1 × 10^−6^ M peptide, then cultured for 10 days in the presence of 25 IU/ml IL-2 (Miltenyi biotech). Specific cells were labeled with a fluorescent tetramer, comprising the relevant peptide and HLA (TCMetrix) and single-cell sorted using a MoFlow cytometer. Clones were expanded with pooled, autologous healthy donor PBMC as feeder cells, PHA-L (Sigma), and 600 IU/ml IL-2. After approximately 20 days, 1–10 × 10^3^ clones were restimulated in the presence of autologous PBMC as feeder cells, PHA-L, and 600 IU/ml IL-2, as described above. Clone specificity was confirmed by tetramer staining.

T cell clones/lines were cultured in RPMI 1640 media supplemented with 20 mM *N*-2-hydroxyethylpiperazine-*N*’-2-ethanesulfonic acid, 60 mg/L penicillin, 12.5 mg/L streptomycin, 2 mM l-glutamine, 1% non-essential amino acids, and 10% human serum (TCRPMI).

### T cell killing assays

Twenty thousand melanoma cells were plated in wells of a flat bottom 96-well plate. T cells were added to selected wells as appropriate to give the effector to target (E:T) ratios shown in the text. Samples were incubated overnight (~16–24 h) at 37°C. The following day, T cells were resuspended by gentle pipetting and then removed. The plate was washed once with PBS. MTS reagent (CellTiter 96^®^ AQueous One Solution Cell, Promega) was added and samples incubated for 1–2 h at 37°C. Absorbance at 490 nm was measured, and normalized to melanoma samples incubated in absence of T cells for each cell line to give percentage of viable cells.

## Results

**Figure 1:**
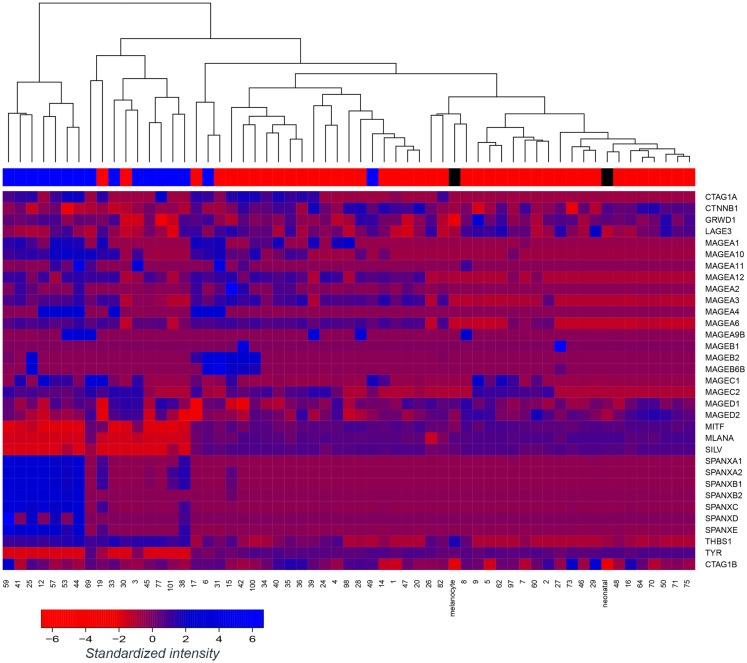
**Tumor antigen expression in melanoma epithelial or mesenchymal-like cell lines**. Microarray-based gene expression of a range of melanoma antigens, including several melanoma differentiation antigens and cancer-testis antigens across a panel of 57 early passage melanoma cell lines and 2 controls (normal melanocytes; “melanocyte” and neonatal melanocytes; “neonatal”). Melanoma cell lines were classified as epithelial-like (top bar; red) or mesenchymal-like (top bar; blue) on the basis of dominant E-cadherin or N-cadherin expression, respectively, however, unsupervised clustering of the cell lines was performed without reference to this annotation. Control melanocytes have been labeled in black.

We assessed gene expression in our panel of 57 early passage melanoma cell lines (7, 24). Cell lines were classified as E-like or M-like on the basis of E-cadherin or N-cadherin expression, respectively. We have previously reported widespread variation in antigen expression between E- or M-like phenotypes (8). Here, we focus on the difference in expression of a range of immunogenic antigens, including MDA and CTAg, shown in Figure 1. As previously reported by ourselves and others, expression of MDA (e.g., Melan-A and tyrosinase) and MITF was largely lower in M-like, compared to E-like, cells. In contrast, a number of CTAg (e.g., SPANX family members) were upregulated in M-like cells. The expression of some antigens (e.g., MAGED1) was unaffected by the differences in phenotype between our cell lines. These data highlight the immunogenic heterogeneity of melanoma.

**Figure 2:**
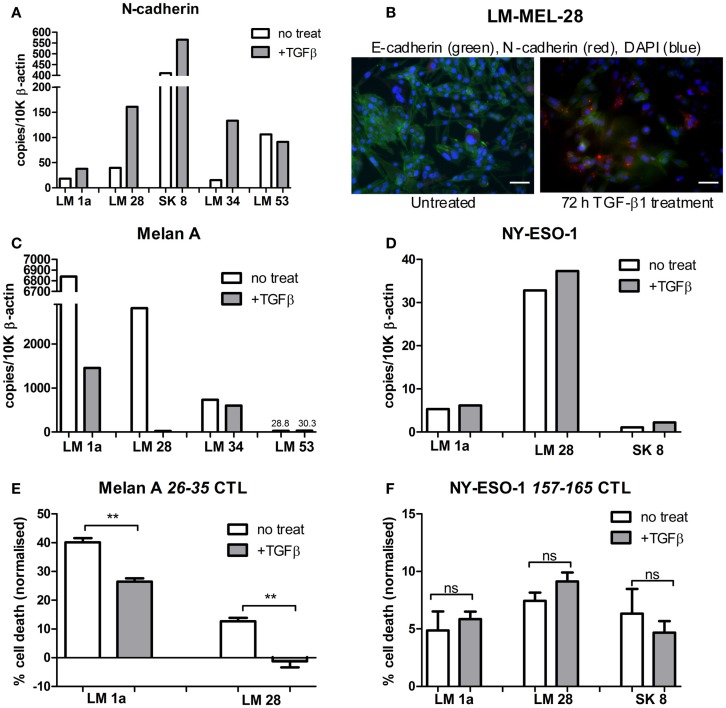
**Antigen expression and T cell-mediated lysis of melanoma cell lines following TGFβ induced EMT**. Epithelial-like melanoma cells lines, LM Mel 1a, LM Mel 28, SK Mel 8, and LM Mel 34, and mesenchymal-like cell line LM Mel 53, were incubated with 5 ng/ml TGFβ for 72 h to induce EMT. Cells were lysed, and cDNA was generated (as in Section “Materials and Methods”). **(A)** Expression levels of N-cadherin in treated and untreated cell lines were determined by qPCR, in order to assess switching to the mesenchymal-like phenotype, and expressed relative to 10,000 copies of β-actin. **(B)** Immunofluorescence confirmed increase in N-cadherin protein expression (Red) and concomitant reduction in E-cadherin (Green) after 72 h of TGF-β1 treatment, shown here for a representative cell line (LM-Mel 28). Scale bar = 100 μM. Expression levels of **(C)** Melan-A and **(D)** NY-ESO-1 were compared between TGFβ treated/untreated melanoma cell lines expressing these antigens. Melanoma cell lines expressing Melan-A, and/or NY-ESO-1 were treated with/without 5 ng/ml TGFβ for 72 h. Specific T cell clones, which recognized the HLA-A*0201 epitopes **(E)** Melan-A 26–35 or **(F)** NY-ESO-1 *157–165* were incubated with 2 × 10^4^ treated/untreated melanoma cells at a 1:1 effector to target ratio. Cells were incubated for 24 h at 37°C. T cells were washed off, followed by addition of MTS reagent (Promega) and 1 h incubation at 37°C. Absorbance was read at 490 nm, and normalized to untreated control cells for each treatment condition. Each measurement was in triplicate, and treated/untreated samples were compared using a paired *T-*test. ns = not significant. **P ≤ 0.01.

Based on our previous studies (7, 8), we selected four E-like melanoma cell lines, which were HLA-A*0201^+^, and which were also positive for expression of either Melan-A, or NY-ESO-1, or both. As a control, we also obtained an M-like cell line (LM-Mel 53). The selected lines were treated with TGFβ for 72 h to induce EMT, and successful switch to an M-like phenotype was confirmed by demonstrating upregulation of N-cadherin gene expression by qPCR, or protein expression by immunofluorescence (Figures 2A,B). In all four E-like cell lines, upregulation of N-cadherin expression was observed following TGFβ incubation, whereas there was no significant change in N-cadherin expression by the M-like cell line.

Epithelial-like cell lines LM-Mel 1a, LM-Mel 28, and LM-Mel 34 express Melan-A under steady state conditions, while the M-like cell line LM-Mel 53 expresses low levels of this antigen. In our heatmap (Figure 1), no cell line demonstrated expression of NY-ESO-1/CTAG1B, which is likely due to inefficiency of the probe (probes to CTAg are traditionally inferior), since we have shown by qPCR (Figure 2D) and IHC (not shown) that several of our cell lines express NY-ESO-1. The effect of phenotypic switching by EMT on the expression of either Melan-A or NY-ESO-1 was assessed in the relevant cell lines. qPCR demonstrated that while gene expression levels of NY-ESO-1 did not alter significantly following EMT, the expression levels of Melan-A decreased in all E-like, but not M-like cell lines expressing this antigen (Figures 2C,D). This result is in line with previous studies from our group and others, which demonstrated downregulation of several MDA including Melan-A and related antigens under the transcriptional control of MITF.

Previous studies have shown that TGFβ treatment results in downregulation of MHC class II molecules, such as HLA-DR; however, no significant effect of TGFβ treatment on HLA class I levels has been demonstrated (25). We assessed HLA Class I expression in our cell lines before and after 72 h TGFβ treatment, and confirmed that no significant change in HLA Class I levels occurred (data not shown). This result is important for our subsequent experiments, which assess T cell killing of TGFβ-treated/untreated melanoma cell lines. In these studies, we can confirm that HLA does not play a limiting role for either melanoma phenotype.

Since M-like cells are generally more resistant to various treatment modalities, we tested whether T cell-mediated lysis of these cells was also impeded. T cells, which recognized the HLA-A*0201 restricted epitope *157–165* from NY-ESO-1 or *26–35* from Melan-A were incubated with melanoma cell lines expressing their respective target antigen, at a 1:1 effector:target ratio. Cells were incubated for 24 h before viability of melanoma cells was assessed using an MTS assay (methods), and normalized to untreated control cells (Figures 2E,F). In all cell lines tested, NY-ESO-1 specific T cells killed melanoma cell lines comparably both before and after TGFβ treatment. In contrast, Melan-A specific T cells were significantly more effective at killing E-like melanoma cells prior to induction of EMT. Following TGFβ treatment, cell lines were less efficiently cleared by Melan-A specific T cells in all cases tested.

Our results indicate that the ability of M-like cells to escape from T cell-meditated lysis was not due to any inherent functional characteristic of these cells, but rather due to changes in expression of target antigen. Melan-A expression decreased following EMT, leading to corresponding diminishing ability of antigen specific T-lymphocytes to kill target cells. In contrast, the expression of NY-ESO-1 was unchanged following EMT, and in parallel the ability of specific T cells to recognize and kill melanoma cells in an antigen specific manner was unaffected.

## Discussion

Contemporary melanoma therapy is undergoing a revolution due to the unprecedented success of immune checkpoint inhibitors; agents that re-activate anti-cancer immunity within treated patients (26). These successes are a culmination of what scientific researchers have long striven to achieve using methods such as cancer vaccination and adoptive cell transfer, that is, appropriate immune targeting in a manner which results in tumor clearance. Phenotype switching by melanoma cells to an M-like phenotype has been considered an inherent mechanism of tumor escape from therapy and resistance to various treatment modalities. Set against the back drop of recent immune-based treatment successes, we find here that immune-mediated clearance of melanoma cells is unaffected by EMT under the appropriate settings.

Previous treatment strategies have been hampered by the heterogeneity of the disease – escape of residual cells from radiotherapy, emergence of resistance to BRAF inhibition, etc – and a strategy which could target the tumor as a whole has eluded researchers. Patients successfully treated by immune checkpoint inhibition can continue to remain disease free long-term [4 years at the most recent follow up for anti-CTLA4 therapy (27), and ~25 months for anti-PD-1 treatment (28)]. It is therefore clear that appropriate immune control can achieve clearance of melanoma.

Despite the relative success of immune checkpoint inhibition, many patients are still not rendered disease free with these agents. In this cohort, rational combination therapies are likely to be beneficial and extensive studies are currently underway to ascertain which kinds of combination will exert broadest patient benefit and disease control. These combination therapies will include targeting melanoma specific antigens by cancer vaccination or adoptive cell therapy, in conjunction with immune checkpoint inhibition, and indeed this is the focus of several current clinical trials (NCT01176474, NCT02077114, NCT01176461, NCT01988077, NCT01701674), including our own trial combining vaccination with NY-ESO-1 and ipilimumab (NCT01810016).

Our study here, and others (8, 19, 29) highlight not only functional but also immune heterogeneity of melanoma cells. We demonstrate that EMT in melanoma can result in escape from T cell targeting. Importantly, we have shown that the means of tumor escape from immune-mediated killing is not due to an intrinsic functional resistance mechanism of cells that display the M-like phenotype, but rather is due to decrease in the expression of target antigen. T lymphocyte targeting of selected antigens with constant expression during EMT results in comparable killing of melanoma cells *in vitro* independent of their phenotype. Since M-like cells have traditionally been considered more difficult to kill than their E-like counterparts, this finding is significant. The ability of M-like and E-like tumor cells to be equally killed by T cells recognizing appropriate targets confirms the potential of combination treatments using appropriately selected antigens as targets.

These data further highlight the importance of antigen selection for cancer vaccination or adoptive cell transfer, since even if completely effective, therapeutic options which target antigens preferentially expressed by E-like cells will be incapable of clearing the tumor as a whole. Indeed such therapies could potentially exert selective pressure for tumor cells with the more aggressive M-like phenotype and thereby promote cell populations that carry a poorer prognosis for the patient.

To develop this rationale further, antigen specific immunotherapy strategies should ideally target antigens which are not only expressed in differentiated E-like cells, but also those which are either induced when the phenotype changes during EMT, or which are consistently expressed in both E-like and M-like cells. The former have been little examined in melanoma to date, most studies focusing on antigen loss leading to immune escape during EMT. However, Figure 1 demonstrates that a subset of MAGE antigens and the SPANX family of CTAgs are upregulated in M-like melanoma cell lines. Furthermore, Boisgerault et al. demonstrated that in prostate cancer, topoisomerase IIα and CD44 are antigens which are specific to M-like cells, and further showed that a subset of melanoma cells also expressed topoisomerase IIα (29, 30).

Hopefully, combination treatment strategies with appropriately selected target antigens as discussed here will result in enhanced tumor clearance irrespective of tumor heterogeneity. Indeed, we are currently assessing the efficacy of this approach using the CTAgs SPANX, expression of which is induced during EMT, and Ropporin, whose expression in melanoma is widespread and constant during EMT (manuscript in preparation).

## Author Contributions

Katherine Woods designed the work, acquired and analyzed data, and wrote the manuscript; Anupama Pasam, Aparna Jayachandran, and Miles C. Andrews acquired and analyzed data and revised the manuscript; Jonathan Cebon contributed to the concept of the work, revised, and approved the manuscript for publication.

## Conflict of Interest Statement

Katherine Woods, Anupama Pasam, and Jonathan Cebon have received funding for unrelated projects from GlaxoSmithKline (GSK). GSK have been developing cancer vaccines based on the cancer testis antigens MAGEA3 and NY-ESO-1.
